# Amyloid-β related angiitis presenting as eosinophilic meningitis: a case report

**DOI:** 10.1186/s12883-022-02638-w

**Published:** 2022-03-24

**Authors:** Jeremy A. Tanner, Megan B. Richie, Cathryn R. Cadwell, Amity Eliaz, Shannen Kim, Zeeshan Haq, Nailyn Rasool, Maulik P. Shah, Elan L. Guterman

**Affiliations:** 1 Department of Neurology, University of California, San Francisco (UCSF), 505 Parnassus Avenue, M798 Box 0114, San Francisco, CA 94143 USA; 2 Department of Anatomic Pathology, University of California, San Francisco (UCSF), San Francisco, CA USA; 3 School of Medicine, University of California, San Francisco (UCSF), San Francisco, CA USA; 4 Department of Ophthalmology, University of California, San Francisco (UCSF), San Francisco, CA USA

**Keywords:** Amyloid-β related angiitis, Eosinophilic meningitis, Cerebral amyloid angiopathy-related inflammation, Primary angiitis of the central nervous system

## Abstract

**Background:**

Eosinophilic meningitis is uncommon and often attributed to infectious causes.

**Case presentation:**

We describe a case of a 72-year-old man who presented with subacute onset eosinophilic meningitis, vasculitis, and intracranial hypertension with progressive and severe neurologic symptoms. Brain MRI demonstrated multifocal strokes and co-localized right temporo-parieto-occipital vasogenic edema, cortical superficial siderosis, and diffuse leptomeningeal enhancement. He ultimately underwent brain biopsy with immunohistochemical stains for amyloid-β and Congo red that were extensively positive in the blood vessel walls and in numerous diffuse and neuritic parenchymal confirming a diagnosis of amyloid-β related angiitis. He was treated with immunosuppression with clinical stabilization.

**Conclusions:**

Amyloid-β related angiitis is an underrecognized cause of eosinophilic meningitis that can present fulminantly and is typically responsive to immunosuppression. The presence of eosinophils may provide additional clues to the underlying pathophysiology of amyloid-β related angiitis.

## Background

Eosinophilic meningitis is defined as cerebrospinal fluid (CSF) with ≥10 eosinophils/microliter or eosinophils comprising ≥10% of the total CSF leukocyte count [1]. Eosinophilic meningitis is uncommon, often attributed to infectious causes, and helps to narrow the differential for meningoencephalitis [1]. Amyloid-β related angiitis (ABRA) is an autoimmune disease characterized by an inflammatory response to amyloid-β deposits in the walls of small vessels in the cortex and leptomeninges, leading to vasculitic destruction [2, 3]. It is typically responsive to immunosuppression, but not previously associated with eosinophilic meningitis. Here we describe a case of eosinophilic meningitis with intracranial hypertension caused by ABRA. We obtained informed consent to write this report.

## Case presentation

A 72-year-old right-handed man with chronic sinusitis, obstructive sleep apnea, and hyperlipidemia presented with 3 weeks of progressive headache, altered mental status, right retro-orbital pain, and blurred vision. Initial evaluation by an optometrist revealed a left homonymous hemianopsia. On the drive home from his appointment, he hit the left road median and was brought to an emergency department. There he underwent head CT and was diagnosed with sinusitis. Despite antibiotics, over the next week he developed disorientation, memory impairment, slowed speech, and a refractory headache, prompting re-presentation.

In the preceding months, he had travelled to Mexico, Panama, and Costa Rica with exposure to mosquitos and a freshwater lake. He worked for an air conditioning company. He occasionally smoked tobacco, drank two glasses of alcohol nightly, and had no illicit drug use. He had no significant family history.

Brain MRI without contrast demonstrated multifocal acute infarcts in the right parieto-occipital region with associated focal cortical superficial siderosis, and a right temporal mass-like lesion with associated mild vasogenic edema. Brain and neck vessel imaging and echocardiogram were unrevealing. He left against medical advice. As an outpatient, an otolaryngologist performed an unrevealing nasal endoscopy.

**Fig. 1:**
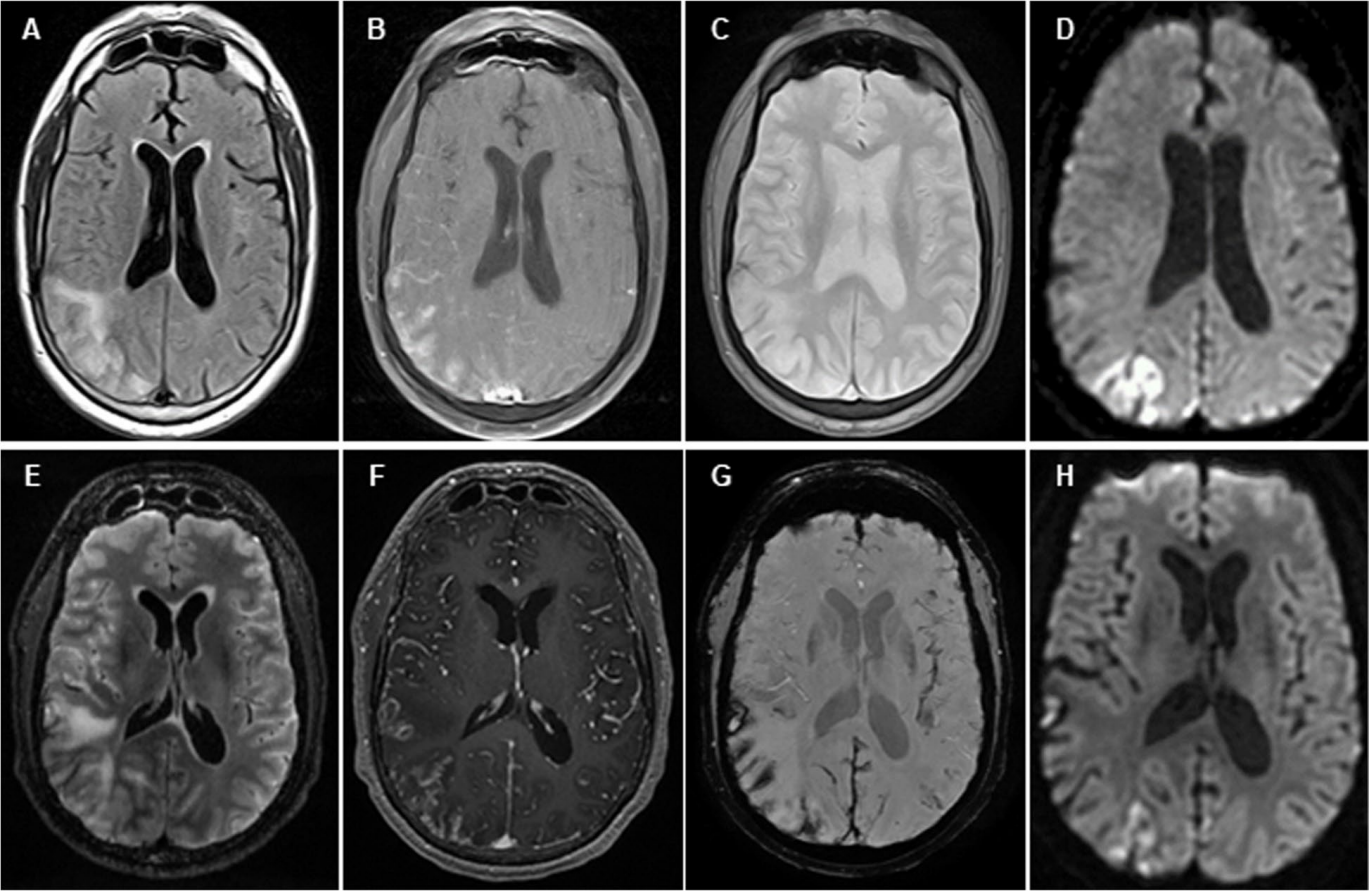
MRI Studies. Top row: MRI obtained on hospital re-presentation, 1 month after symptom onset. There is co-localized right temporo-parieto-occipital T2 hyperintensity with vasogenic edema (**A**), leptomeningeal enhancement on T1 post-contrast (**B**), and cortical superficial siderosis on T2 gradient echo sequences (**C**). Multifocal right parieto-occipital (**D**) and bilateral cerebellar punctate (not shown) infarcts were present on diffusion-weighted imaging. Bottom Row: MRI obtained on hospital transfer 1 week later and prior to first brain biopsy. There is T2 hyperintensity with vasogenic edema in the right temporo-parietal lobe and FLAIR non-suppression (**E**) with associated diffuse leptomeningeal enhancement on T1 post-contrast (**F**) and diffuse cortical superficial siderosis on susceptibility weighted imaging (**G**). Multifocal right parieto-occipital (**H**) and new left frontal punctate (not shown) infarcts were present on diffusion-weighted imaging

His symptoms progressed and he re-presented a week later with rapidly progressive dementia, now unable to care for himself. Serum workup was unremarkable. CSF revealed white blood cell (WBC) count 20 cells/microliter (4% eosinophils, 72% lymphocytes, 24% monocytes), protein 1.75 g/L, glucose 3.1 mmol/L, and red blood cell (RBC) count 140 cells/microliter. Brain MRI with and without contrast (Fig. 1A-D) demonstrated multifocal strokes and co-localized right temporo-parieto-occipital vasogenic edema, cortical superficial siderosis, and diffuse leptomeningeal enhancement. He was empirically started on broad-spectrum antibiotics and antivirals and transferred to our facility.

**Fig. 2:**
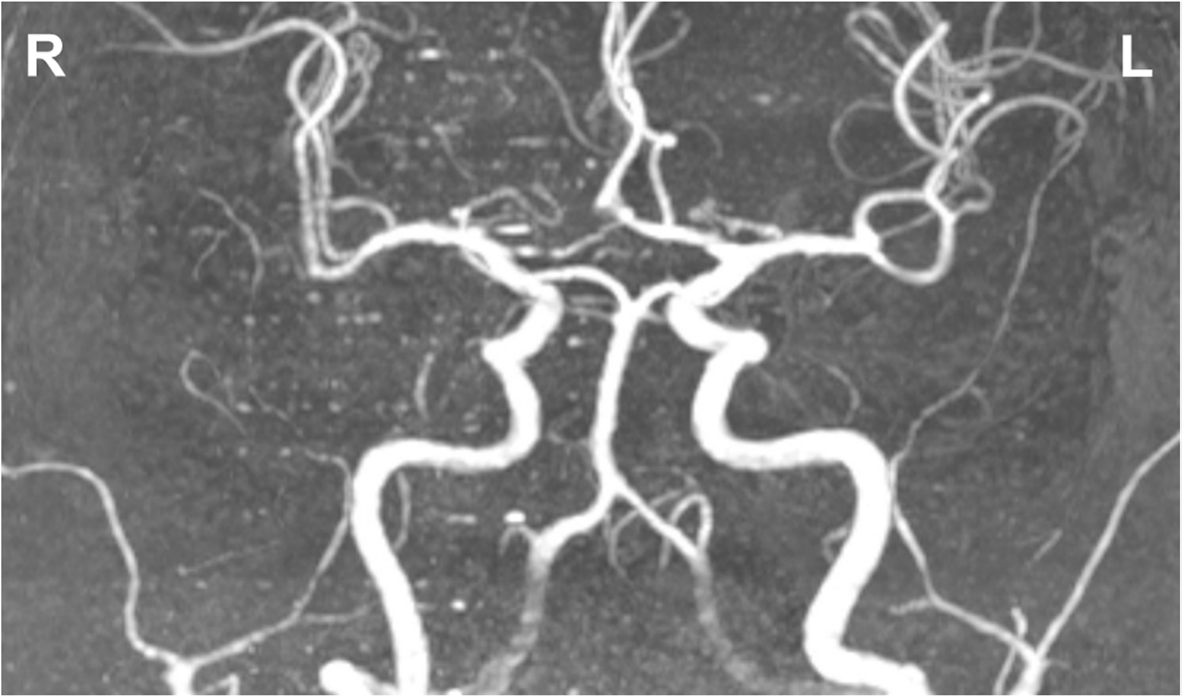
MR Angiography. Imaging obtained after hospital transfer and prior to first brain biopsy. The number and caliber of right middle cerebral artery branch vessels were diminished

On initial exam, vital signs were normal. He had meningismus, somnolence, inattention, and executive dysfunction. He had a mild left hemiparesis, left homonymous hemianopsia, and bilateral optic nerve swelling with peripapillary hemorrhages. Serum studies demonstrated no leukocytosis or eosinophilia. Lumbar puncture revealed a markedly elevated opening pressure of 46 cm H_2_O, worsened eosinophilia (WBC 14 cells/microliter: 19% eosinophils, 65% lymphocytes, 16% monocytes), protein 2.25 g/L, glucose 3.5 mmol/L, 2 unique oligoclonal bands, immunoglobulin G index 1.0, and benign cytology and flow cytometry. MRI brain with and without contrast (Fig. 1E-H) showed progression. MR angiography showed diminished number and caliber of right middle cerebral artery branch vessels without large vessel occlusions or irregularities (Fig. 2). Conventional cerebral angiography was not obtained. He had extensive negative serum and CSF testing for bacterial, viral, fungal, mycobacterial, and parasitic causes of eosinophilic meningoencephalitis including angiostrongyliasis, baylisascariasis, neurocysticercosis, universal polymerase chain reaction, and metagenomic next generation sequencing. Broad evaluation for autoimmune and neoplastic disorders, including whole-body CT and fluorodeoxyglucose-PET, were unrevealing. Video-EEG for 72 h revealed diffuse severe slowing and asymmetric right-sided, posterior-predominant voltage attenuation suggestive of focal cerebral pathology, aligning with imaging findings. There were no epileptiform discharges or seizures.

He underwent biopsy of a region of enhancement and edema in his right lateral temporal lobe and leptomeninges. Pathology showed a dense inflammatory infiltrate involving the leptomeninges and underlying cortex, composed predominantly of lymphocytes and plasma cells with a smaller component of eosinophils. No granulomatous inflammation or vasculitis was present. Gram, Periodic Acid Schiff, Grocott’s methenamine-silver, Steiner silver, and Kinyoun acid-fast stains were negative for bacterial, fungal, spirochete, and acid-fast organisms. No parasites were seen. Congo Red stain was negative.

**Fig. 3:**
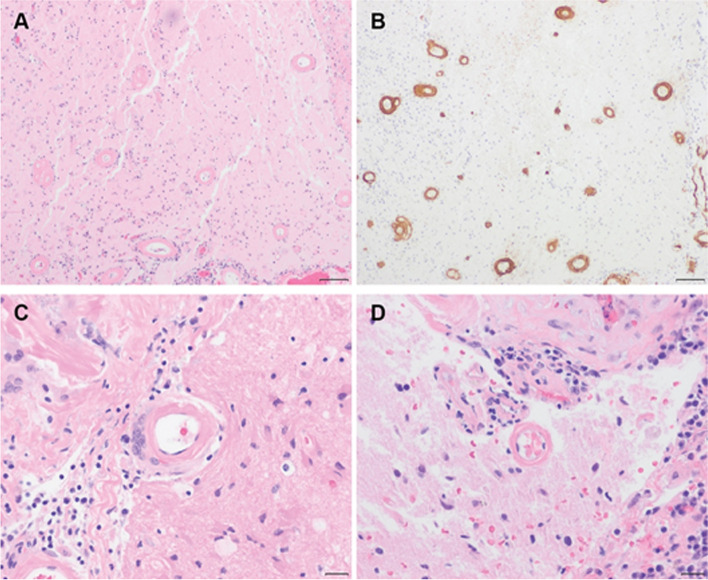
Pathological Studies. Brain biopsy demonstrating morphologic and immunohistochemical features of amyloid-β related angiitis (ABRA). Hematoxylin and eosin-stained sections revealed marked thickening of the leptomeningeal and cortical blood vessel walls by a homogenous eosinophilic material (**A**) which was positive for amyloid-β by immunohistochemistry (**B**). Scattered blood vessels also showed perivascular granulomatous inflammation with multinucleated giant cells (**C**) and transmural lymphoplasmacytic inflammation with fibrinoid necrosis of the blood vessel wall (**D**). Images were taken using an Olympus BX51 microscope equipped with 10× (0.30 NA) and 40× (0.75 NA) objectives and a Zeiss AxioCam HRc camera using ZEN 3.0 software with a resolution of 1.4 and 0.34 μm/pixel for panels **A–B** and **C–D**, respectively. Any adjustments were applied to the entire image. Threshold manipulation, expansion or contraction of signal ranges, and altering of high signals were not performed. Scale bars are 100 μm (**A** and **B**) and 20 μm (**C** and **D**)

He became obtunded and continued to worsen radiographically, prompting initiation of empiric antifungal, antiparasitic, and high-dose steroid therapy. He had serial lumbar punctures for intracranial hypertension. Diagnostically, he underwent a second brain biopsy targeting worsening enhancement and edema in the right occipital lobe. Biopsy revealed thickening of the small and medium caliber blood vessels within the leptomeninges and cortex by a homogenous eosinophilic material (Fig. 3A-B). Additionally, granulomatous and transmural lymphoplasmacytic inflammation of several blood vessels with focal vessel wall necrosis were seen, consistent with angiitis (Fig. 3C-D). Immunohistochemical stains for amyloid-β and Congo red were extensively positive in the blood vessel walls and in numerous diffuse and neuritic parenchymal plaques.

He was diagnosed with ABRA and received 5 days of high-dose methylprednisolone with a prolonged taper and pulsed intravenous cyclophosphamide. His clinical course stabilized, his intracranial hypertension resolved, and his MRI enhancement resolved. His exam stabilized but only marginally improved. At 9 months follow-up, he remained dependent in activities of daily living, reliant on a feeding tube, and bedbound.

## Discussion and conclusions

This case report describes fulminant eosinophilic meningitis and vasculitis with multifocal strokes and intracranial hypertension caused by ABRA. To our knowledge, this is the first reported case where the eosinophilic pleocytosis is associated with intracranial hypertension resulting in papilledema, and the third reported case of eosinophilic meningitis due to ABRA [4, 5]. In the first case in the literature, the patient presents with a lobar hemorrhage prompting consideration of cerebral amyloid angiopathy (CAA), then re-presents with subacute cognitive decline and aphasia [4]. The second case is described as eosinophilic meningitis due to primary angiitis of the central nervous system (PACNS), although the biopsy demonstrates a diagnosis of ABRA [5]. Our case further emphasizes that ABRA should be added to the differential diagnosis of eosinophilic meningitis (Table 1), while also demonstrating the variable clinical presentation of the disease by describing an unusual and severe syndrome. In all three cases, eosinophilia was isolated to the CSF, helping to distinguish ABRA from other causes of eosinophilic meningitis that commonly include peripheral eosinophilia [1]. Additionally, all three patients had stabilization but not resolution of symptoms at the time of treatment, illustrating the importance of an early diagnosis. ABRA is important to include in the differential for eosinophilic meningitis because, unlike infectious causes which may worsen with immunosuppression, ABRA is typically responsive to immunosuppression and most patients have a positive outcome if treated early [3, 6, 7].

**Table 1:** Causes of Eosinophilic Meningitis^a^

Infectious:	Noninfectious:
**Parasitic**	**Autoimmune**
Angiostrongyliasis	Neuromyelitis Optica^b^
Baylisascariasis	*Amyloid-β Related Angiitis (ABRA)*^b^
Gnathostomiasis	Sarcoidosis (rare)
Neurocysticercosis	Granulomatosis with Polyangiitis (rare)
Paragonimiasis	**Neoplastic**
Schistosomiasis	Hodgkin’s Lymphoma^b^
Toxocariasis	Non-Hodgkin’s Lymphoma (rare)^b^
**Fungal**	Leukemia^b^
Coccidioidomycosis^b^	Other tumors with meningeal spread^b^
Cryptococcosis (rare)^b^	**Hypereosinophilic Syndrome**
Allergic Aspergillus Sinusitis (rare)^b^	**Chemical Meningitis**
**Other Infectious**	Ventriculoperitoneal shunt^b^
*Rickettsia rickettsii* (rare)^b^	Myelography contrast^b^
	Medications (rare)^b^ – ibuprofen, ciprofloxacin, gentamicin, vancomycin, sulfamethoxazole/trimethoprim

^a^Other possible causes (exceedingly rare or limited evidence): neurosyphilis, tuberculosis, coxsackie virus, lymphochoriomeningitis virus, visceral myiasis, trichinellosis, echinococcosis, fascioliasis, strongyloidiasis, rheumatoid arthritis, Behcet’s disease, illicit intravenous drug use

^b^Typically lack peripheral blood eosinophilia

ABRA is characterized by an inflammatory response to amyloid-β deposits in the walls of small vessels in the cortex and leptomeninges, leading to granulomatous transmural inflammation and vasculitic destruction of the vessel wall [2, 3]. A related pathological subtype, often referred to as “inflammatory CAA” or “cerebral amyloid angiopathy-related inflammation (CAARI),” has been described when there is perivascular inflammation around amyloid-β deposits in vessels, but without vessel wall destruction [3, 6, 8]. The terminology for these entities is often used interchangeably in the literature [9]. Thus far, clinically meaningful differences have not been identified between these pathological subtypes, and they may represent varying severities of the same disease [3, 6]. Alternatively, PACNS is distinguished by transmural inflammation and vessel wall destruction without associated amyloid-β deposits, and has been associated with a higher risk of ischemic stroke and a lower risk of intracerebral hemorrhage [3]. CAA is distinguished by amyloid-β deposits in vessel walls without associated inflammation and is not treated with immunosuppression. ABRA is thought to exist in a spectrum between CAA and PACNS, and to be more clinically similar to PACNS than to CAA [3, 4, 8].

The progressive deposition of amyloid-β in the walls of small arteries, arterioles, and capillaries in the cortex and leptomeninges in CAA appears to be largely due to impaired clearance of amyloid-β, particularly through perivascular drainage pathways [10–12]. In cerebral arteries, amyloid-β deposits in basement membranes in the tunica media, then replaces smooth muscles cells, and eventually replaces all elements in the arterial walls [11, 12]. Growing evidence suggests that ABRA is driven by an autoimmune response against these vascular amyloid-β deposits [13, 14]. Similar radiographic findings termed amyloid-related imaging abnormalities (ARIA) have been reported in up to 40% of participants in clinical trials of monoclonal antibody therapies targeting amyloid-β, such as bapineuzumab and aducanumab [15, 16]. As anti-amyloid therapies enter routine clinical practice, understanding the different clinical syndromes associated with amyloid-related autoimmunity will be increasingly important. Investigators hypothesize that anti-amyloid therapy causes amyloid-β plaque solubilization, leading to greater stress on amyloid-β clearance pathways from brain parenchyma and subsequent amyloid-β deposition in vessel walls. These vascular amyloid-β deposits then trigger an immune response in some patients [2, 15]. ABRA may involve a similar pathogenesis, except inflammation is triggered spontaneously rather than by anti-amyloid therapy [2, 13, 14, 17]. The role of eosinophils in this process is unknown but may reflect (1) an immune response to amyloid-β vascular deposits [4]; (2) an immune response to hemosiderin [18, 19]; or (3) an attempt to facilitate tissue repair [18].

ABRA typically presents at a mean age of 67 and symptoms progress over 6 months, although it can present acutely (≤2 days) in a quarter of cases [3, 7]. Common symptoms include altered mental status (75%), headaches (32%) and seizures (32%). Other symptoms include weakness, aphasia, and visual changes [7].

Diagnostic workup commonly reveals normal serum studies. CSF is inflammatory with mild-to-moderate elevated protein and mild lymphocytic or mixed pleocytosis, although it may be eosinophilic [2, 7]. Radiographically, ABRA should be considered anytime there is co-localized (1) T2 hyperintensity with vasogenic edema that is commonly bilateral and asymmetric; (2) microhemorrhages or superficial siderosis; and (3) focal leptomeningeal enhancement [3, 6]. There may also be infarcts and mass-like lesions [3].

To avoid brain biopsy, clinicoradiological diagnostic criteria have been proposed for CAARI based on common clinical (i.e., headache, decrease in consciousness, behavior change, focal neurologic signs) and imaging (i.e., white matter hyperintensities, hemorrhagic lesions) features and the exclusion of other potential causes [20]. These criteria were validated in a small sample of 17 participants with CAARI and 37 participants with CAA with up to 82% sensitivity and 97% specificity. In cases that meet the criteria, a clinicoradiological diagnosis may help to expedite treatment without the need for biopsy [20] These criteria can presumably also be used to distinguish ABRA from CAA, although this requires further validation.

Recently developed amyloid-β biomarkers may also support the diagnosis of ABRA and CAA by enabling the detection of amyloid-β molecular pathology without biopsy. Patients with ABRA have decreased CSF amyloid-β_42_ compared to controls [21]. Additionally, amyloid-β PET scans have been positive in multiple case reports of patients with ABRA, and shown to have a high sensitivity for CAA [8, 17, 22, 23]. As the prevalence of amyloid PET positivity increases with age in cognitively unimpaired controls, a positive amyloid PET scan would be particularly supportive of the diagnosis in younger patients when there is a clinical suspicion for ABRA [24]. Preliminary studies have identified CSF autoantibodies to amyloid-β that decrease with treatment, showing promise as a potential future biomarker for ABRA, but these are not yet clinically available [13, 17, 25].

Brain biopsy remains the gold standard for diagnosis, particularly when the diagnosis is unclear and alternative causes cannot be excluded. Importantly, a negative biopsy should not exclude the diagnosis. The histopathologic changes in CAA, ABRA, and PACNS are often focal, commonly yielding false-negative biopsy results due to the patchy pattern of amyloid-β deposition in vessels and the segmental and skip-lesion nature of vascular lesions in cerebral vasculitis [12, 26, 27]. False-negative biopsy results have been identified in up to 35% of cases of PACNS, though the frequency is unknown in ABRA [27]. Due to this, we suspect the initial biopsy in our case was falsely negative, and the second biopsy was diagnostic.

There are multiple notable features in this case. First, ABRA should be recognized as a treatable cause of eosinophilic meningitis. Second, intracranial hypertension was prominent with associated obtundation and papilledema, and later improved with serial lumbar punctures and steroids. ABRA has previously been associated with elevated intracranial pressure and severe vasogenic edema, independent of large infarcts, in approximately 12% of cases and can progress rapidly to death [2, 28]. Third, final diagnosis required obtaining a biopsy from the occipital lobe, an area in which amyloid-β in CAA predominates [22]. In cases of possible ABRA, biopsy should target contrast-enhancing posterior regions, and include leptomeninges, gray matter, and white matter. Fourth, this case demonstrated an example of co-localized cortical superficial siderosis, leptomeningeal enhancement, and T2 hyperintensity on imaging early in presentation, which should prompt consideration of ABRA.

Contrary to infectious causes of eosinophilic meningitis, ABRA is treated with immunosuppression using high-dose steroids and/or cyclophosphamide. Over half of patients have a favorable response to treatment with mild or no disability after 24-month follow-up, and ABRA has significantly greater survival than CAA [3, 6, 7]. However, approximately 16% have moderate to severe disability and 29% die despite treatment [7]. Earlier treatment is presumably associated with better outcomes by preventing permanent ischemic brain injury. Considering ABRA in the differential diagnosis of eosinophilic meningitis may lead to earlier diagnosis and treatment to prevent disability in future patients.

## Data Availability

Data sharing is not applicable to this article as no datasets were generated or analyzed during the current study. The authors are available to answer any questions related to this case that follows Health Insurance Portability and Accountability Act privacy protection.

## Abbreviations

MRI: Magnetic resonance imaging
CSF: Cerebrospinal fluid
ABRA: Amyloid-β related angiitis
CT: Computed tomography
WBC: White blood cell
RBC: Red blood cell
PET: Positron emission tomography
EEG: Electroencephalography
PACNS: Primary angiitis of the central nervous system
CAA: Cerebral amyloid angiopathy
